# *Lactobacillus plantarum* lipoteichoic acid inhibits biofilm formation of *Streptococcus mutans*

**DOI:** 10.1371/journal.pone.0192694

**Published:** 2018-02-08

**Authors:** Ki Bum Ahn, Jung Eun Baik, Ok-Jin Park, Cheol-Heui Yun, Seung Hyun Han

**Affiliations:** 1 Department of Oral Microbiology and Immunology, DRI, and BK21 Plus Program, School of Dentistry, Seoul National University, Seoul, Republic of Korea; 2 Research Division for Biotechnology, Korea Atomic Energy Research Institute, Jeongeup, Republic of Korea; 3 Department of Agricultural Biotechnology and Research Institute for Agriculture and Life Sciences, Seoul National University, Seoul, Republic of Korea; University of Florida, UNITED STATES

## Abstract

Dental caries is a biofilm-dependent oral disease and *Streptococcus mutans* is the known primary etiologic agent of dental caries that initiates biofilm formation on tooth surfaces. Although some *Lactobacillus* strains inhibit biofilm formation of oral pathogenic bacteria, the molecular mechanisms by which lactobacilli inhibit bacterial biofilm formation are not clearly understood. In this study, we demonstrated that *Lactobacillus plantarum* lipoteichoic acid (Lp.LTA) inhibited the biofilm formation of *S*. *mutans* on polystyrene plates, hydroxyapatite discs, and dentin slices without affecting the bacterial growth. Lp.LTA interferes with sucrose decomposition of *S*. *mutans* required for the production of exopolysaccharide, which is a main component of biofilm. Lp.LTA also attenuated the biding of fluorescein isothiocyanate-conjugated dextran to *S*. *mutans*, which is known to have a high affinity to exopolysaccharide on *S*. *mutans*. Dealanylated Lp.LTA did not inhibit biofilm formation of *S*. *mutans* implying that D-alanine moieties in the Lp.LTA structure were crucial for inhibition. Collectively, these results suggest that Lp.LTA attenuates *S*. *mutans* biofilm formation and could be used to develop effective anticaries agents.

## Introduction

A biofilm is a dense community of bacteria attached to an organic or inorganic surface. Generally, bacteria in the biofilm are enclosed in an extracellular polymeric substance matrix of polysaccharides, proteins, extracellular DNA, and metabolites [1]. During the transition from planktonic to biofilm bacteria, a variety of physiological characteristics change. Genes associated with adhesion molecules or exopolysaccharide (EPS) and antibiotic-resistance genes are increased in biofilm bacteria [2]. Bacteria in biofilms are known to be 10- to 1000-times more resistant to antibiotics and antimicrobial peptides than planktonic bacteria. Biofilm bacteria can avoid phagocytosis by macrophages or neutrophils [3, 4]. Accordingly, biofilms are a public health concern due to increased resistance to antibiotics that limits treatment options.

Dental caries is a representative biofilm-associated infectious disease of the oral cavity accompanied by tooth acidification and demineralization [5]. Cariogenic bacteria such as *Streptococcus mutans*, *Streptococcus sobrinus*, and lactobacilli are important in the pathogenesis of dental caries [6]. Among these bacteria, *S*. *mutans*, a facultative anaerobic Gram-positive bacterium, is a main cause of enamel decay and caries. *S*. *mutans* has characteristics for colonization of hard tissues in the human oral cavity and mediation of cariogenic biofilms through metabolism of dietary sugar. In particular, glucosyltransferases (GTFs) are involved in *S*. *mutans* biofilm formation by creating α1,3- and α1,6-linked glucan chains using carbohydrates such as sucrose and glucose [7]. Glucan mediates the adherence of *S*. *mutans* to tooth surfaces, bacterial cell-to-cell adhesion, and the formation of the extracellular polymeric substance matrix that provides the structural integrity and stability of biofilms.

Commensal bacteria are known to have beneficial effects on hosts. In particular, *Lactobacillus* species that constitute a major part of microbiota interfere with infection with pathogens by producing antimicrobial molecules, improving epithelial barrier function, and inhibiting adherence of pathogens to epithelial cells [8, 9]. Lactobacilli also have beneficial effects on oral health. For example, *Lactobacillus acidophilus* culture supernatants alleviate gingivitis and periodontitis [10]. *Lactobacillus plantarum*, *Lactobacillus reuteri*, and *Lactobacillus rhamnosus* GG inhibit *S*. *mutans* biofilm formation [11]. However, the mechanisms by which lactobacilli inhibit biofilm formation of pathogens responsible for dental caries have not been determined.

Lipoteichoic acid (LTA), a major cell wall component of Gram-positive bacteria, is an amphiphilic glycolipid linked to a hydrophilic polyphosphate polymer [12]. LTA is known to be involved in bacterial growth, resistance to cationic antimicrobial peptides, bacterial adhesion, biofilm formation, and stimulation of host immunity [12, 13]. Lactobacilli LTA is reported to interfere with infection by pathogens. For example, *L*. *plantarum* LTA inhibits production of pro-inflammatory cytokines, chemokines, and endotoxin shock induced by *Shigella flexneri* and *Vibrio anguillarum* [14–17]. *L*. *plantarum* LTA induces production of anti-inflammatory cytokines without inducing inflammatory responses [16]. However, the regulatory effect of lactobacilli LTA on *S*. *mutans* biofilm formation that is closely associated with dental caries is rarely studied. Therefore, in this study, we investigated the inhibitory effect and the underlying molecular mechanisms of LTA from lactobacilli strains on *S*. *mutans* biofilm formation.

## Materials and methods

### Bacteria and reagents

*L*. *plantarum* KCTC10887BP and *S*. *mutans* KCTC 3065 were obtained from the Korean Collection for Type Culture (Daejeon, Republic of Korea). *S*. *mutans* Ingbritt, OMZ-65, and LM-7 were obtained from the Korean Collection for Oral Microorganisms (Seoul National University, Seoul, Republic of Korea). Clinical strains of *S*. *mutans* KCOM1197 and KCOM1214 were obtained from the Korean Collection for Oral Microbiology (Chosun University, Gwangju, Republic of Korea). Fluorescein isothiocyanate-conjugated dextran (dextran-FITC) and LIVE/DEAD Bacterial Viability Kits were from Molecular Probes (Eugene, OR, USA). Muramyl dipeptide (MDP) and L-ala-gamma-D-glu-mDAP (Tri-DAP) were from InvivoGen (San Diego, CA, USA). Proteinase K and octyl-sepharose CL-4B beads were from Sigma-Aldrich Chemical Inc. (St. Louis, MO, USA).

### Purification of LTA

LTA was prepared from *L*. *plantarum* (Lp.LTA) as previously described [18]. Structural intactness of Lp.LTA was confirmed with high-field nuclear magnetic resonance and matrix-assisted laser desorption ionization-time of flight mass spectrometry as previously described [19]. LTAs purified from *Lactobacillus sakei* K101, *Lactobacillus delbrueckii* K552, and *L*. *rhamnosus* GG ATCC53103 were kindly provided by Prof. Dae Kyun Chung at Kyung Hee University (Suwon, Republic of Korea). No biologically active molecules such as endotoxins, nucleic acids, or proteins were detected in the purified LTA preparations [19–21]. Lp.LTA with D-alanine removed was prepared by incubating intact Lp.LTA with 0.1 M Tris-HCl at pH 8.5 for 24 h [22]. Dealanylation of LP.LTA was confirmed with thin layer chromatography using 1% ninhydrin solution and 5% phosphomolybdic acid.

### Preparation of lipoproteins and peptidoglycan

Lipoproteins from *L*. *plantarum* were isolated as described previously [23]. Bacterial pellets were harvested and suspended in Tris-buffered saline (TBS) containing proteinase inhibitors. Bacterial lysate was incubated with 2% Triton X-114 at 4°C for 2 h. After centrifugation, supernatants were collected and further incubated at 37°C for 15 min. After centrifugation, aqueous phase was discarded and an equal volume of TBS was added to the Triton X-114 phase. After incubating at 37°C for 15 min, the Triton X-114 phase was collected by centrifugation and mixed with methanol at -20°C overnight. Precipitated lipoproteins were dissolved in 10 mM octyl β-D-glucopyranoside. Peptidoglycan from *L*. *plantarum* was isolated as described previously [24, 25]. Bacteria pellets were disrupted by a bead beater and centrifuged at 800 x *g* for 10 min to remove cell debris. Supernatants were recentrifuged at 20,000 x *g* for 10 min and pellets were heated at 60°C for 30 min in 0.5% sodium dodecyl sulfate (SDS). Precipitates were treated with 10 μg/ml DNase, 50 μg/ml RNase, and 200 μg/ml trypsin at 37°C for 18 h. After centrifugation, pellets were suspended in 5% trichloroacetic acid and incubated at 26°C for 18 h. Precipitates were treated with pre-chilled acetone that was removed by washing with pyrogen-free water. Final pellets were collected as purified peptidoglycan suspended in pyrogen-free water.

### Preparation of hydroxyapatite discs

Hydroxyapatite discs (10 mm diameter, 2 mm thickness) were prepared by sintering of hydroxyapatite powder (Sigma-Aldrich Chemical Inc.) as previously described [26]. Hydroxyapatite powders were pressed at 3000 MPa by using a hydraulic press (Carver, IN, USA). Compressed hydroxyapatite powders were sintered at 1200°C for 24 h using an electric furnace (Korea Furnace, Seoul, Republic of Korea). To confirm sterility, hydroxyapatite discs were incubated in brain-heart infusion (BHI) broth (BD Bioscience, Franklin Lakes, NJ, USA) overnight at 37°C and incubated media plated on BHI agar plates. Bacterial colonies were not observed at the end of the incubation period. Hydroxyapatite discs were coated with human saliva prior to use [27].

### Preparation of human dentin slices

Preparation and use of human dentin slices was approved by the Institutional Review Board of Seoul National University Dental Hospital, Seoul, Republic of Korea (CRI 15007). The institutional review board waived the written informed consent from study subjects. Single-rooted premolars with fully formed apices were obtained from patients undergoing extractions for orthodontics in the Department of Oral and Maxillofacial Surgery at Seoul National University Dental Hospital. Calculus and soft tissue on the root surfaces were removed by an ultrasonic scaler. Cleaned teeth were sliced into 500 μm thick cross sections using an Isomet precision saw (Buehler, Lake Bluff, IL, USA). Dentin slices were treated with 17% EDTA for 5 min, followed by treatment with 2.5% sodium hypochlorite for 5 min. After neutralizing with 5% sodium thiosulfate for 5 min, dentin slices were autoclaved for 15 min at 121°C. To confirm sterility, dentin slices were incubated in BHI broth overnight at 37°C and the incubated media were plated on BHI agar plates. Bacterial colonies were not observed at the end of the incubation period. Dentin slices were coated with human saliva prior to use [28].

### Measurement of biofilm formation using crystal violet staining

*S*. *mutans* (1 × 10^8^ CFU/ml) was grown in 96-well plates at 37°C for 24 h in BHI medium supplemented with 0.05% sucrose. After incubation, planktonic bacteria were removed by gentle washing with phosphate buffered saline (PBS), and biofilms were stained with 0.1% crystal violet solution for 30 min at room temperature. Plates were rinsed with PBS and adhered dye was dissolved with a solution (95% ethanol and 0.1% acetic acid in water). Absorbance was measured at 600 nm with a microplate reader (Molecular Devices, CA, USA) and the results were expressed as the percentage of control group.

### Measurement of biofilm formation using confocal laser scanning microscopy

*S*. *mutans* (1 × 10^8^ CFU/ml) was grown in glass bottom dishes at 37°C for 24 h in BHI medium supplemented with 0.05% sucrose. After incubation, planktonic bacteria were removed by gentle washing with PBS, and bacterial biofilms were determined with LIVE/DEAD Bacterial Viability Kits containing SYTO9 and propidium iodide according to the manufacturer's instructions. The excitation/emission maxima were 480/500 nm for SYTO9 and 490/635 nm for propidium iodide. Fluorescence was visualized with LSM700 confocal laser scanning microscope (Zeiss, Jena, Germany). Simultaneous dual channel imaging was used to display green and red fluorescence.

### Scanning electron microscopy

Scanning electron microscope analysis was performed as described previously [29]. *S*. *mutans* (1 × 10^8^ CFU/ml) was grown in 24-well plates, or on saliva-coated HA discs or dentin slices at 37°C for 24 h in BHI medium supplemented with 0.05% sucrose. Adherent bacteria were prefixed with a PBS containing 2.5% glutaraldehyde and 2% paraformaldehyde (pH 7) at 4°C overnight and washed with PBS. Samples were subsequently fixed with 1% osmium tetroxide for 90 min, washed three times with distilled water, and dehydrated by replacing buffer with increasing concentrations of ethanol (70%, 80%, 90%, 95%, and 100% for 15 min each). After drying with hexamethyldisilazane and coating with ion sputter, samples were observed under a scanning electron microscope (S-4700, Hitachi, Tokyo, Japan).

### Measurement of EPS by flow cytometry

*S*. *mutans* (1 × 10^8^ CFU/ml) was grown in the presence or absence of Lp.LTA in BHI medium supplemented with 0.05% sucrose at 37°C for 24 h. After incubation, Lp.LTA was removed by gentle washing with PBS three times, and EPS of *S*. *mutans* was stained with 5 μg/ml of dextran-FITC for 10 min. The production of EPS was determined by flow cytometry using FACSCalibur with CellQuest software (BD Biosciences, San Diego, CA, USA). Percentage of EPS-positive *S*. *mutans* was shown in each histogram.

### High-performance liquid chromatography

Sucrose decomposition by enzymatic reaction of *S*. *mutans* was determined by high-performance liquid chromatography with refractive index detector (HPLC-RID) on the Agilent 1200 Infinity LC system (Agilent Technologies, Boeblingen, Germany). *S*. *mutans* (1 × 10^8^ CFU/ml) was grown in BHI medium supplemented with 0.5% sucrose at 37°C for 24 h in the presence or absence of Lp.LTA. Then, culture supernatants were obtained and subjected to HPLC-RID analysis. Separation of the samples was performed on the Aminex HPX-87P column with distilled water as the mobile phase and eluted in isocratic mode at a flow rate of 0.5 ml/min for 25 min. The injection volume was 10 μl. Data acquisition and analysis were performed by using the Agilent ChemStation software (Agilent Technologies).

### Statistical analysis

All experiments were performed at least three times. Mean ± standard deviation (S.D.) was obtained from triplicate samples for each treatment group. Statistical significance was examined using *t*-test and results were considered significant at *P* < 0.05.

## Results

### *L*. *plantarum* LTA inhibits *S*. *mutans* biofilm formation

**Fig 1:**
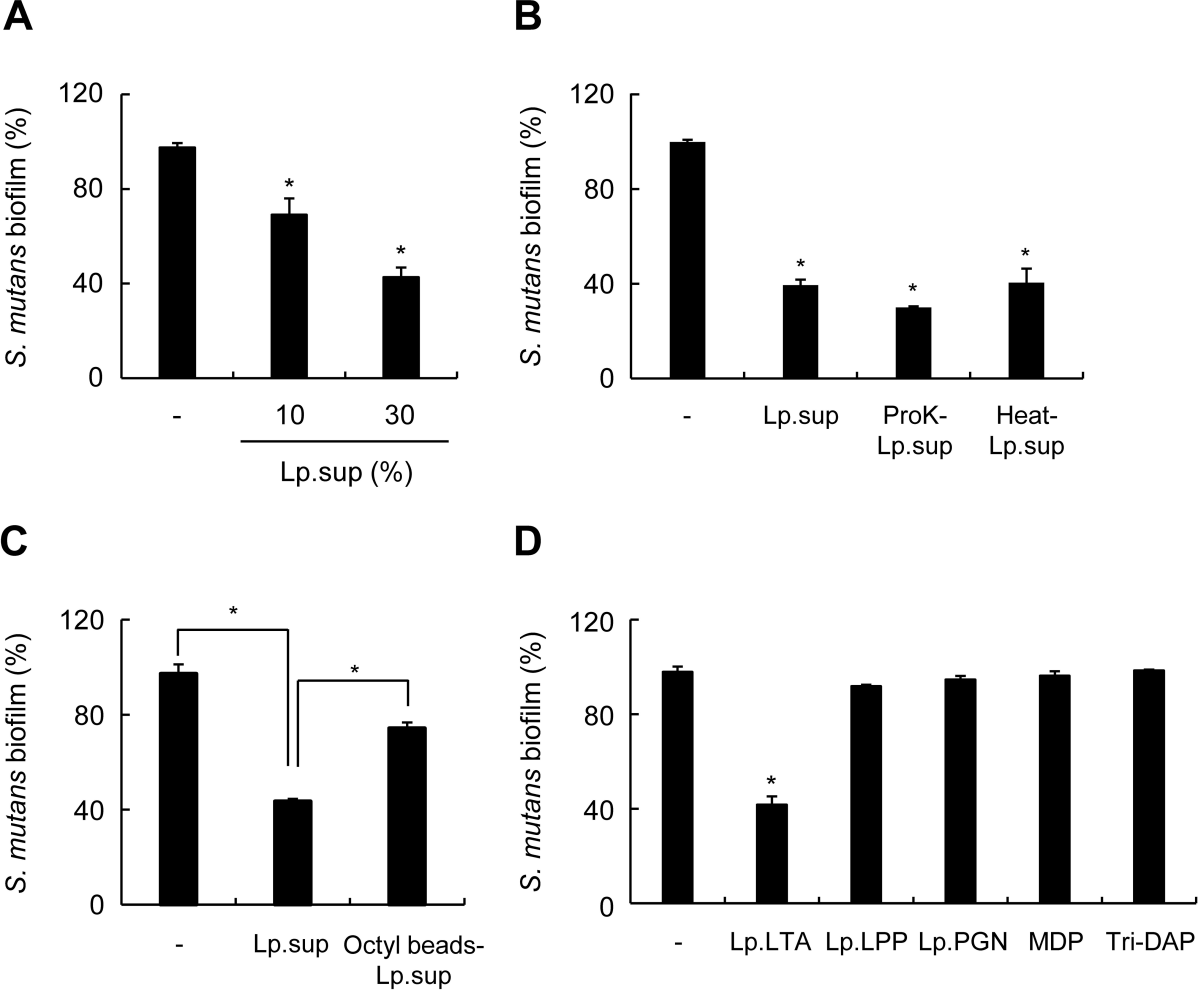
*L*. *plantarum* LTA inhibits *S*. *mutans* biofilm formation. *S*. *mutans* (1 × 10^8^ CFU/ml) was grown on 96-well polystyrene plates at 37°C for 24 h in the presence or absence of (A) *L*. *plantarum* culture supernatant (Lp.sup) at the indicated concentrations; (B) 20% of Lp.sup, proteinase K-treated Lp.sup (ProK-Lp.sup), or heat-treated Lp.sup (Heat-Lp.sup); (C) 20% of Lp.sup or octyl-sepharose beads-treated Lp.sup (Octyl beads-Lp.sup); (D) 30 μg/ml of *L*. *plantarum* LTA (Lp.LTA), *L*. *plantarum* lipoprotein (Lp.LPP), *L*. *plantarum* peptidoglycan (Lp.PGN), MDP, or Tri-DAP. Biofilm formation extent was determined by a crystal violet assay. Data are mean values ± S.D. of triplicate samples. Asterisk, significant induction at *P* < 0.05 compared with non-treatment control group.

Culture supernatants of lactobacilli such as *L*. *plantarum*, *Lactobacillus helveticus*, and *L*. *acidophilus*, inhibit microbial biofilm [11, 30]. We examined the effect of *L*. *plantarum* culture supernatant (Lp.sup) on *S*. *mutans* biofilm formation. Lp.sup inhibited *S*. *mutans* biofilm formation in a dose-dependent manner (Fig 1A). To characterize molecules that might be involved in Lp.sup-mediated inhibition of *S*. *mutans* biofilm formation, *S*. *mutans* was treated with Lp.sup, proteinase K-treated Lp.sup, heat-treated Lp.sup, or Lp.sup treated with octyl-sepharose beads. Proteinase K or heat treatment hardly altered the inhibitory effects of Lp.sup on the biofilm formation of *S*. *mutans* (Fig 1B), whereas Lp.sup treated with octyl-sepharose beads remarkably recovered inhibition of *S*. *mutans* biofilm formation (Fig 1C). These results suggest that some hydrophobic molecules are essential components in Lp.sup for inhibiting *S*. *mutans* biofilm formation. To identify molecule(s) in Lp.sup that inhibited *S*. *mutans* biofilm formation, *S*. *mutans* was treated with microbe-associated molecular patterns from bacterial culture supernatants such as *L*. *plantarum* LTA (Lp.LTA), *L*. *plantarum* lipoproteins (Lp.LPP), *L*. *plantarum* peptidoglycan (Lp.PGN), MDP, or Tri-DAP. Lp.LTA inhibited biofilm formation of *S*. *mutans*, whereas the other molecules did not affect *S*. *mutans* biofilm formation (Fig 1D). These results suggest that Lp.LTA might be a key molecule in Lp.sup responsible for inhibition of *S*. *mutans* biofilm formation.

### LTA purified from *L*. *plantarum* inhibits biofilm formation and aggregation of *S*. *mutans* in dental biofilm models

**Fig 2:**
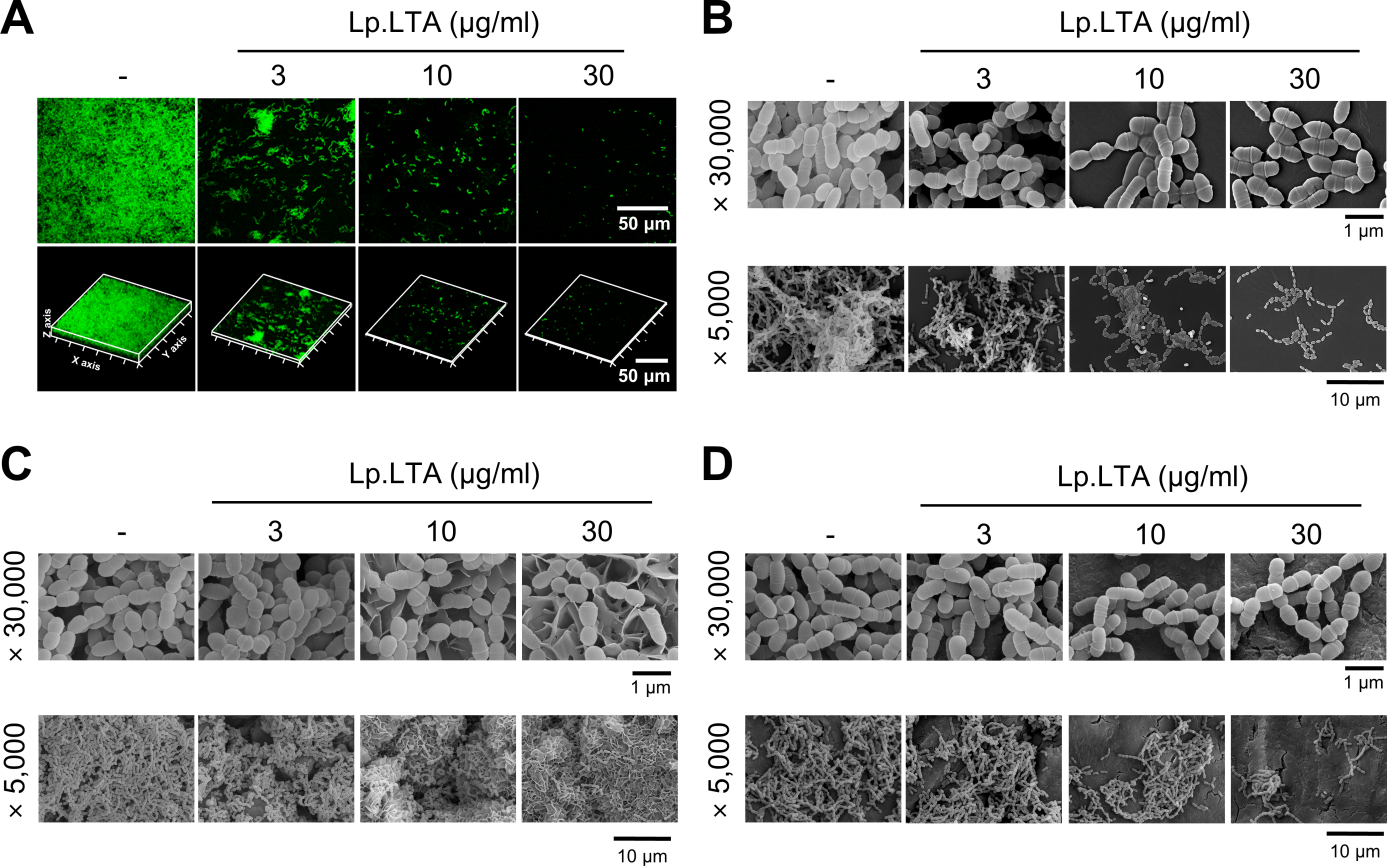
*L*. *plantarum* LTA inhibits biofilm formation and aggregation of *S*. *mutans* in dental biofilm models. (A) *S*. *mutans* (1 × 10^8^ CFU/ml) was grown in glass bottom dishes at 37°C for 24 h in the presence or absence of Lp.LTA at 3, 10, or 30 μg/ml. Lp.LTA-treated *S*. *mutans* biofilms were observed by confocal laser scanning microscopy (green, SYTO9; red, propidium iodide). (B-D) *S*. *mutans* (1 × 10^8^ CFU/ml) was grown on (B) 24-well polystyrene plates, (C) saliva-coated hydroxyapatite discs, or (D) saliva-coated dentin slices at 37°C for 24 h in the presence or absence of Lp.LTA at 3, 10, or 30 μg/ml. Lp.LTA-treated *S*. *mutans* biofilms were visualized by scanning electron microscopy (magnification: × 5,000 and × 30,000).

*S*. *mutans* biofilm formation in the presence of Lp.LTA was evaluated by using confocal laser scanning microscopy and scanning electron microscopy. *S*. *mutans* biofilm formation and aggregation were inhibited by Lp.LTA in a dose-dependent manner (Fig 2A and 2B). *S*. *mutans* is involved in dental caries and apical periodontitis through forming biofilms [6, 31]. To investigate the inhibitory effect of Lp.LTA against *S*. *mutans* biofilm formation in dental biofilm models representing dental caries and apical periodontitis, *S*. *mutans* biofilm on saliva-coated hydroxyapatite discs or human dentin slices was examined after Lp.LTA treatment. Lp.LTA inhibited *S*. *mutans* biofilm formation on both models in a dose-dependent manner (Fig 2C and 2D). Notably, a different morphology in Fig 2C seems to be due to the particular surface structure of hydroxyapatite discs. These results suggest the possibility that Lp.LTA could be used clinically to treat dental infectious diseases such as dental caries or apical periodontitis.

### *L*. *plantarum* LTA, but not other *Lactobacillus*-derived LTAs, inhibits the biofilm of *S*. *mutans* clinical isolates

**Fig 3:**
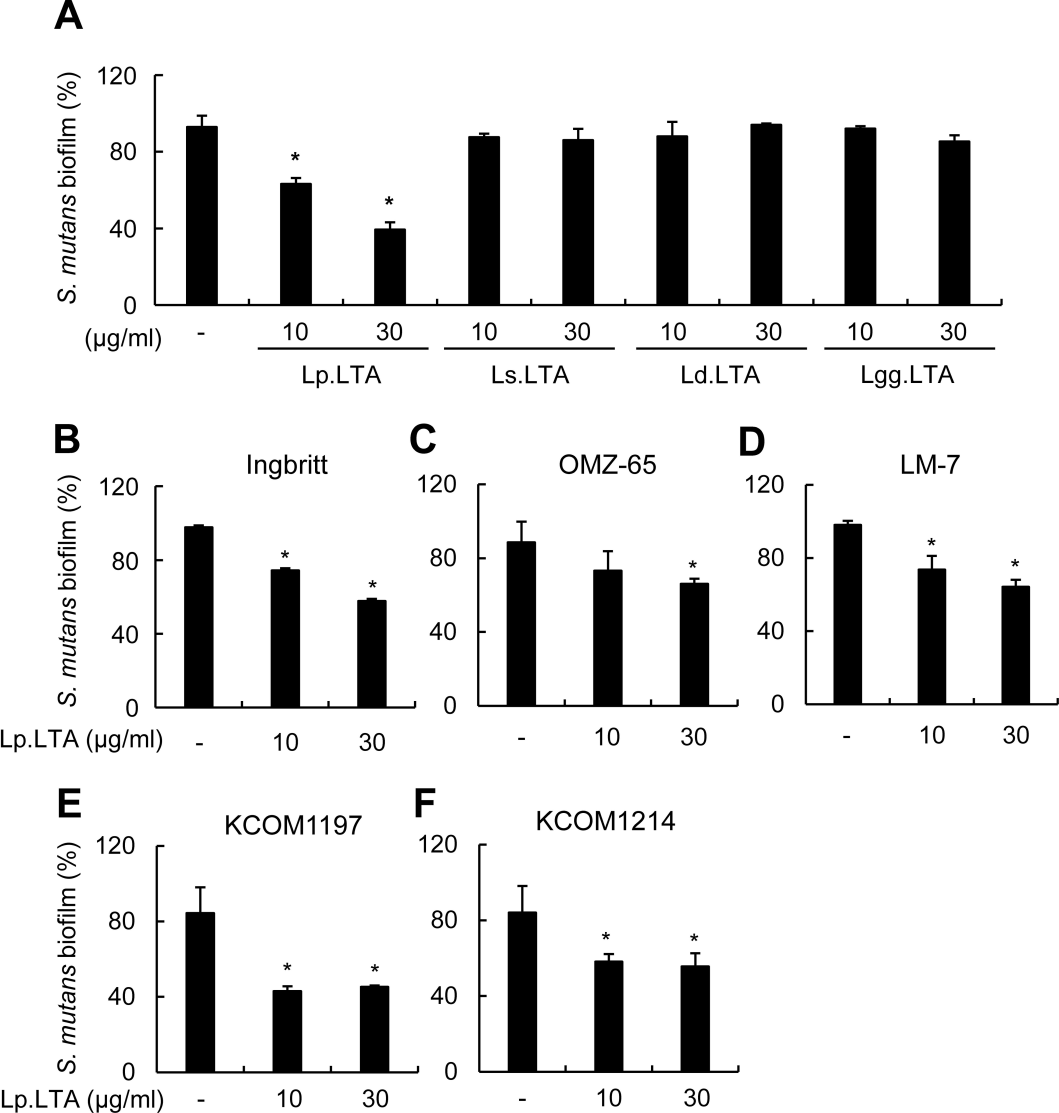
*L*. *plantarum* LTA inhibits biofilm formation of various *S*. *mutans* strains. (A) *S*. *mutans* (1 × 10^8^ CFU/ml) was grown on 96-well polystyrene plates at 37°C for 24 h in the presence or absence of Lp.LTA, *Lactobacillus sakei* LTA (Ls.LTA), *Lactobacillus delbrueckii* LTA (Ld.LTA), or *Lactobacillus rhamnosus GG* LTA (Lgg.LTA) at the indicated concentrations. *S*. *mutans* (B) Ingbritt, (C) OMZ-65, (D) LM-7, (E) KCOM1197, or (F) KCOM1214 was cultured on 96-well polystyrene plates at 37°C for 24 h with Lp.LTA at 10 or 30 μg/ml. Biofilm formation extent was determined by the crystal violet assay. Data are mean values ± S.D. of triplicate samples. Asterisks, significant induction at *P* < 0.05 compared with non-treatment control group.

In order to examine if the inhibitory effect was unique to Lp.LTA, *S*. *mutans* biofilm was formed in the presence of LTAs from various *Lactobacillus* species including *L*. *plantarum* (Lp.LTA), *L*. *sakei* (Ls.LTA), *L*. *delbrueckii* (Ld.LTA), and *L*. *rhamnosus* GG (Lgg.LTA). Of the LTAs from tested *Lactobacillus* species, only Lp.LTA showed a significant inhibition of *S*. *mutans* biofilm formation (Fig 3A). To confirm the inhibitory effect of Lp.LTA on biofilm formation of various *S*. *mutans* strains, *S*. *mutans* Ingbritt, OMZ, and LM-7 strains were treated with different doses of Lp.LTA. *S*. *mutans* Ingbritt, OMZ, and LM-7 were substantially inhibited by Lp.LTA (Fig 3B–3D). To determine if Lp.LTA inhibited clinical strains as well as laboratory strains, the clinical isolates *S*. *mutans* KCOM1197 and KCOM1214 were treated with different doses of Lp.LTA. KCOM1197 and KCOM1214 biofilms were efficiently inhibited by Lp.LTA (Fig 3E and 3F). These results suggest that Lp.LTA could be used as a universal therapeutic agent for *S*. *mutans* biofilm-associated dental diseases regardless of strain type.

### Inhibition of *S*. *mutans* biofilm formation by *L*. *plantarum* LTA lasts till late stages of biofilm development without affecting bacterial growth

**Fig 4:**
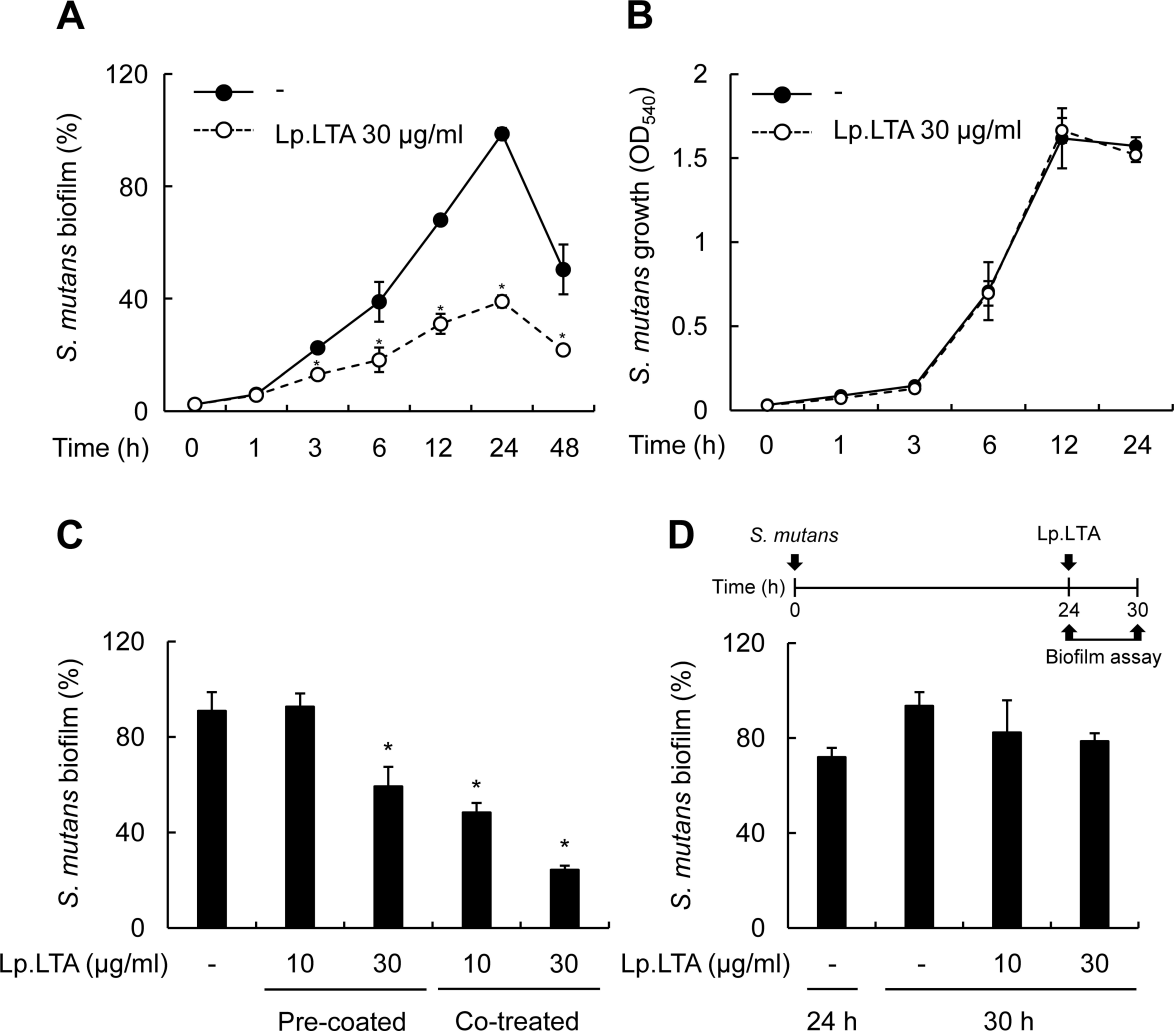
Inhibition of *S*. *mutans* biofilm formation by *L*. *plantarum* LTA lasts till late stages of biofilm development without affecting the bacterial growth. (A) *S*. *mutans* (1 × 10^8^ CFU/ml) was grown on 96-well polystyrene plates at 37°C for 1, 3, 6, 12, 24, or 48 h in the presence or absence of Lp.LTA at 30 μg/ml. Biofilm formation extent was determined by the crystal violet assay. (B) *S*. *mutans* (1 × 10^8^ CFU/ml) was grown at shaking condition for 1, 3, 6, 12, or 24 h in the presence of Lp.LTA at 30 μg/ml. *S*. *mutans* growth was determined by the optical density at 540 nm with a spectrophotometer. (C) *S*. *mutans* (1 × 10^8^ CFU/ml) was grown in the presence of Lp.LTA at 10 or 30 μg/ml on Lp.LTA-uncoated polystyrene plates (Co-treated) or was grown on the plates pre-coated with Lp.LTA at 10 or 30 μg/ml (Pre-coated) at 37°C for 24 h. (D) *S*. *mutans* (1 × 10^8^ CFU/ml) was grown on polystyrene plates at 37°C for 24 h, and then supernatant containing planktonic bacteria was removed. Pre-formed biofilm was treated with Lp.LTA (10 or 30 μg/ml) and further incubated at 37°C for 6 h. Biofilm formation was determined by a crystal violet assay. Data are mean values ± S.D. of triplicate samples. Asterisks, significant induction at *P* < 0.05 compared with non-treatment control group.

To examine if inhibition by Lp.LTA occurred at an early phase of biofilm development or lasted till the late phase, *S*. *mutans* biofilm formation was determined at 1, 3, 6, 12, 24, or 48 h after Lp.LTA treatment. Lp.LTA inhibition of *S*. *mutans* biofilm began to be observed at the early bacterial attachment stage at 3 h and remained till the late mature biofilm formation stage at 48 h (Fig 4A). Next, to examine if inhibition of biofilm formation by Lp.LTA was due to suppression of bacterial growth or survival or to direct inhibition of biofilm formation, *S*. *mutans* was treated with Lp.LTA and incubated for 1, 3, 6, 12, and 24 h at shaking condition. Lp.LTA did not significantly affect *S*. *mutans* growth (Fig 4B). We further examined if the inhibitory effect of Lp.LTA on the biofilm formation was due to interference with the binding of *S*. *mutans* to plastic surface. As shown in Fig 4C, biofilm formation was inhibited by pre-coating the culture plate with Lp.LTA, but to lesser degree, in comparison with the Lp.LTA co-treatment. It implies that the Lp.LTA inhibition of bacterial biding to plastic surface through a simple competition is unlikely to be the major mechanism. Notably, Lp.LTA did not affect the established biofilm (Fig 4D).

### D-Alanine moiety of *L*. *plantarum* LTA is essential for inhibition of *S*. *mutans* biofilm formation

**Fig 5:**
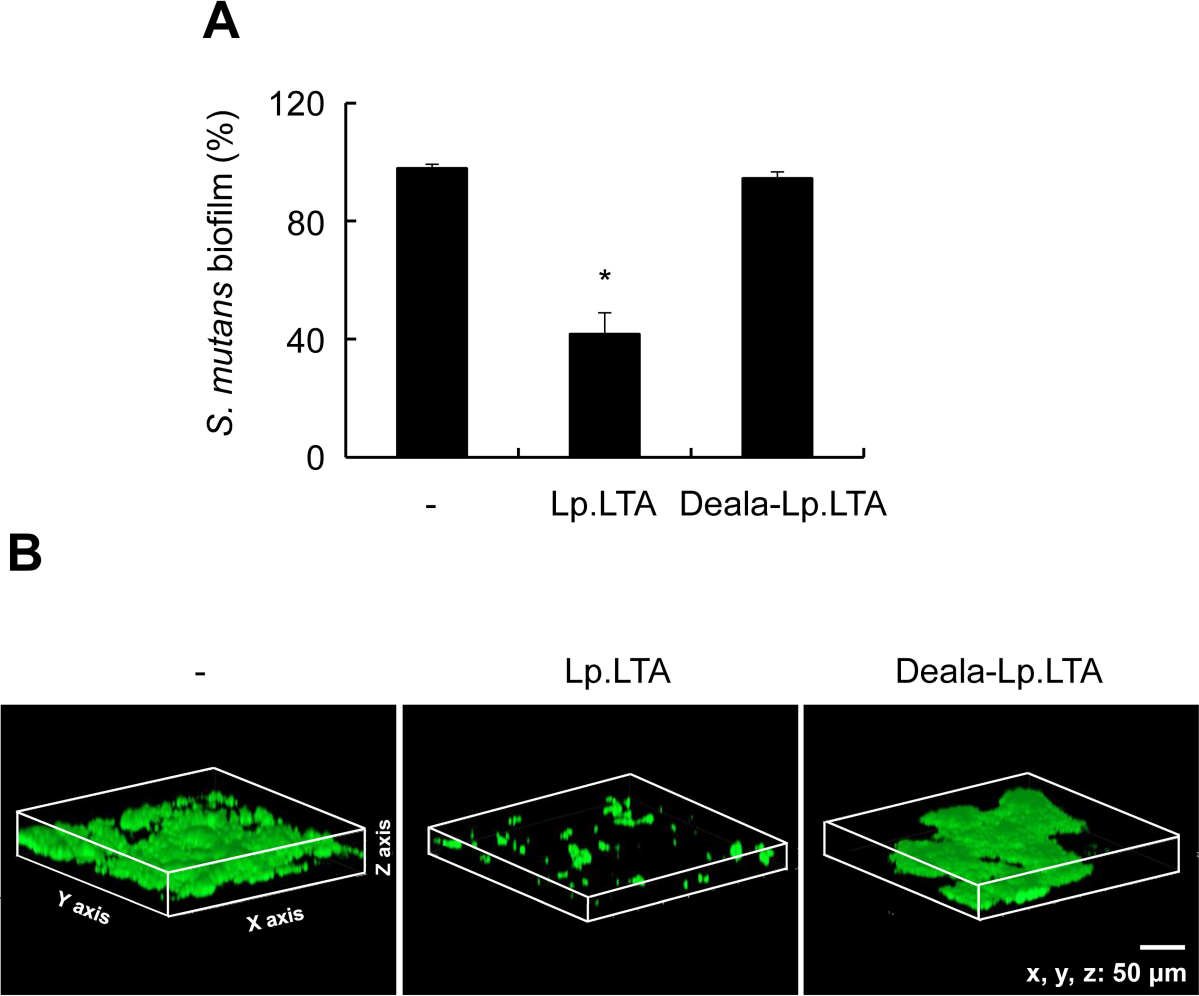
D-Alanine residues are essential for inhibition of *S*. *mutans* biofilm formation by *L*. *plantarum* LTA. (A) *S*. *mutans* (1 × 10^8^ CFU/ml) was grown on 96-well polystyrene plates at 37°C for 24 h in the presence or absence of Lp.LTA and dealanylated Lp.LTA (Deala-Lp.LTA) at 30 μg/ml. Biofilm formation extent was determined by the crystal violet assay. Data are mean values ± S.D. of triplicate samples. Asterisks, significant induction at *P* < 0.05 compared with non-treatment control group. (B) *S*. *mutans* (1 × 10^8^ CFU/ml) was grown on glass bottom dishes at 37°C for 24 h in the presence or absence of Lp.LTA (30 μg/ml) or Deala-Lp.LTA (30 μg/ml). *S*. *mutans* biofilms were observed by confocal laser scanning microscopy (green, SYTO9; red, propidium iodide).

D-Alanine residues are known as functional moieties of LTA involved in the bacterial pathogenesis and the host immunity [12]. To determine the functional moieties required for Lp.LTA inhibition of *S*. *mutans* biofilm formation, *S*. *mutans* was treated with Lp.LTA or dealanylated Lp.LTA (Deala-Lp.LTA). Inhibitory effect of *S*. *mutans* biofilm formation by Lp.LTA was abolished with dealanylated Lp.LTA (Fig 5A). Confocal laser scanning microscopy indicated that *S*. *mutans* biofilm formation was inhibited by Lp.LTA, but not by dealanylated Lp.LTA (Fig 5B). These data indicate that D-alanine moieties are essential for Lp.LTA-induced inhibition of *S*. *mutans* biofilm.

### *L*. *plantarum* LTA interferes with sucrose decomposition and dextran-FITC binding to *S*. *mutans*

**Fig 6:**
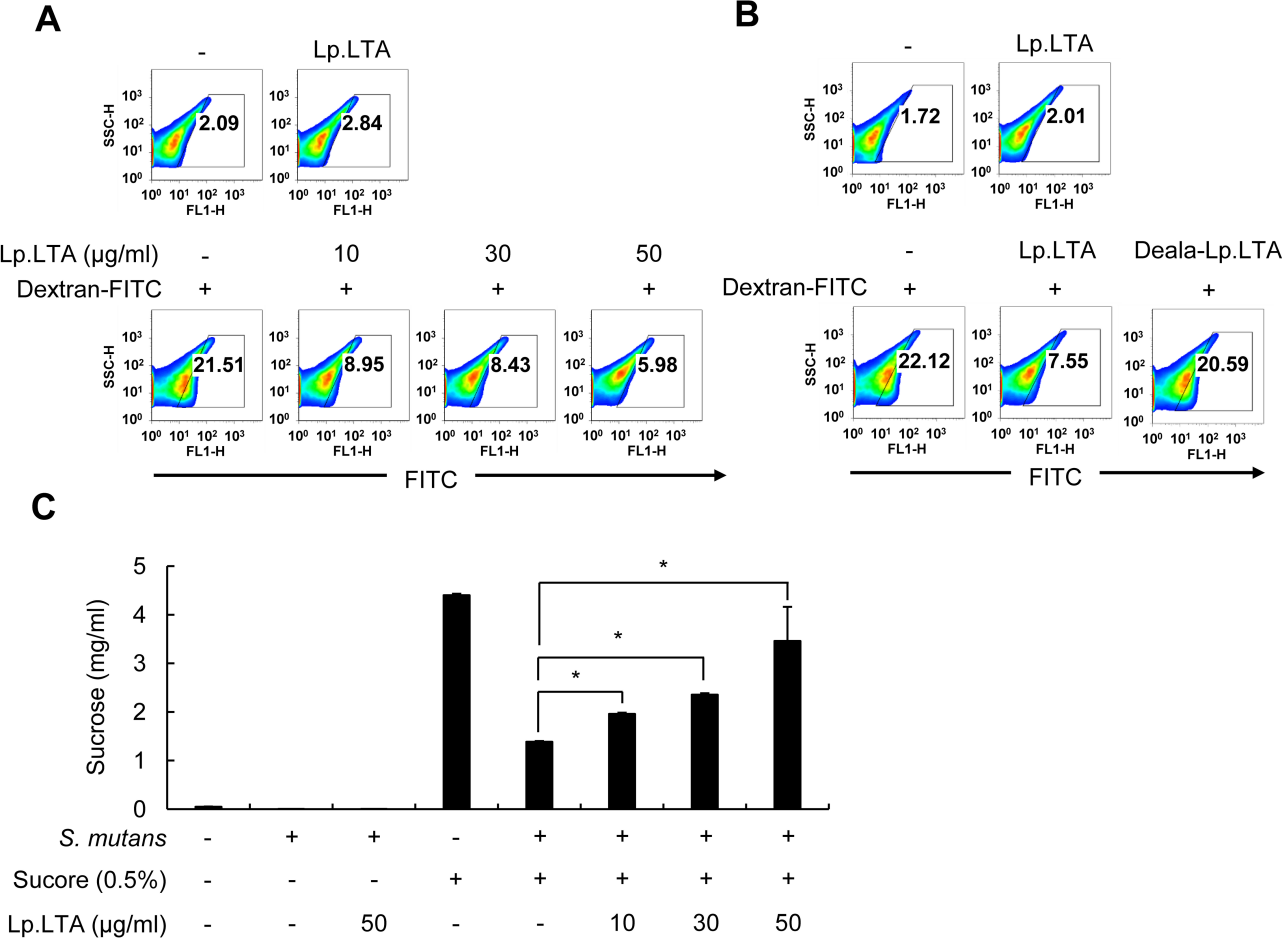
*L*. *plantarum* LTA interferes with sucrose decomposition and dextran-FITC binding to *S*. *mutans*. *S*. *mutans* (1 × 10^8^ CFU/ml) was grown in BHI medium at 37°C for 24 h in the presence or absence of (A) Lp.LTA at the indicated concentrations and (B) Lp.LTA or Deala-Lp.LTA at 30 μg/ml. *S*. *mutans* exopolysaccharide was determined by flow cytometry using dextran-FITC. Percentage of exopolysaccharide-positive *S*. *mutans* is in histograms. One of the three similar results is shown. (C) *S*. *mutans* (1 × 10^8^ CFU/ml) was grown in BHI medium supplemented with 0.5% sucrose at 37°C for 24 h in the presence or absence of Lp.LTA (10, 30, or 50 μg/ml). The culture supernatants were subjected to HPLC-RID for sucrose detection. Data are mean values ± S.D. of triplicate samples. Asterisks indicate significant difference at *P* < 0.05 compared with non-treatment control group.

Production of EPS such as glucan is important for initiation and development of *S*. *mutans* biofilms [7]. To examine the effect of Lp.LTA on EPS production by *S*. *mutans*, *S*. *mutans* EPS was measured by flow cytometry with dextran-FITC at 24 h after culturing *S*. *mutans* in the presence or absence of Lp.LTA. Binding of dextran-FITC to *S*. *mutans* was dose-dependently inhibited by Lp.LTA (Fig 6A and 6B). Although we could not rule out the possibility that Lp.LTA may have interfered with dextran-FITC binding to *S*. *mutans*, these results suggest that Lp.LTA attenuates EPS production of *S*. *mutans*. Interestingly, inhibition did not occur with dealanylated Lp.LTA, implying that D-alanine moieties in Lp.LTA are critical for the attenuated EPS production. In addition, to examine the effect of Lp.LTA on sucrose decomposition, the contents of sucrose in culture supernatant of *S*. *mutans* were examined by HPLC-RID after culturing *S*. *mutans* in the presence or absence of Lp.LTA. As shown in Fig 6C, Lp.LTA inhibited sucrose decomposition by *S*. *mutans* in a dose-dependent manner. Thus, Lp.LTA seems to prevent *S*. *mutans* from degradation of sucrose to produce EPS that is required for biofilm formation.

## Discussion

In this study, we demonstrated that LTA released from *L*. *plantarum* inhibited biofilm formation of *S*. *mutans*, a representative pathogen that causes dental caries. Mechanistic studies showed that biofilm formation was inhibited by Lp.LTA and mediated through suppression of EPS production. D-Alanine in Lp.LTA was a key functional moiety for eliciting the inhibition.

Some bacteria have been reported to produce agents that are anti-adhesive for pathogenic microorganisms. For example, biosurfactants from *Lactobacillus fermentum* inhibit *S*. *mutans* biofilms [32], rhamnolipid from *Burkholderia thailandensia* inhibits *Actinomyces naeslandii* biofilms [33], and lipopeptide from *Bacillus subtilis* inhibits *Staphylococcus aureus* [34]. The activity of biosurfactants is reduced by proteinase K treatment [35]. In contrast, our results showed that the effective molecule in *L*. *plantarum* culture supernatants that inhibited *S*. *mutans* biofilm formation was resistant to proteinase K treatment. LTA purified from *L*. *plantarum* was also resistant to proteinase K treatment and effectively inhibited *S*. *mutans* biofilm formation. Our observation was in keeping with previous reports though the source of LTA and target bacteria used in the study are different from ours. For example, *Streptococcus pyogenes* LTA inhibited aggregation of streptococci [36] and *L*. *fermentum* LTA and *S*. *mutans* LTA inhibited adherence of *S*. *mutans* to glass surfaces [37]. Therefore, along with biosurfactants, Lp.LTA might be an antibiofilm agent against *S*. *mutans*. Both biosurfactants and LTA are structurally amphipathic [38, 39]. Thus, hydrophilic and hydrophobic moieties may be involved in inhibition of biofilm formation. The amphipathic molecules are classified to cationic, anionic, non-ionic, and zwitterionic molecules according to the ionic properties of hydrophilic region. Cationic amphiphiles are known to be specialized to inhibit bacterial biofilm formation. The cationic amphiphiles have been reported to interfere with the aggregation of cells by binding to negatively-charged surface of bacteria or to regulate biofilm-related gene expression by binding to bacterial DNA [40, 41], leading to inhibition of biofilm.

EPS such as glucan is known to be involved in *S*. *mutans* biofilm formation, adherence to tooth surfaces, and bacterial cell-cell interactions [42, 43]. We found that dextran-FITC binding to *S*. *mutans* and sucrose decomposition by *S*. *mutans* dose-dependently decreased with Lp.LTA treatment. These results were consistent with a previous report that LTAs of *L*. *fermentum* and *S*. *mutans* inhibit GTF activities that are crucial for glucan production [37]. Moreover, *Streptococcus sanguinis* LTA is reported to inhibit EPS binding to glucan-binding protein (GBP), which is also required for biofilm formation [44]. However, Lp.LTA did not alter the expression of GTF-B, GTF-C, or GBP-C mRNA in our study. This result supported the possibility that Lp.LTA inhibited EPS production by inhibiting the enzymatic activities of GTFs and/or GBP function without interfering with their expression.

We demonstrated that D-alanine residues are essential for inhibition of *S*. *mutans* biofilm formation by Lp.LTA. Accumulating reports suggest that positively-charged D-alanine ester residues in the LTA structure are important for inflammatory responses, adherence, and resistance to antimicrobial peptides [12, 45–47]. Besides, positive charges have been used to prevent biofilm formation. For example, chitosan conjugates to antibiotics enhance antibiofilm activity, especially biofilms of Gram-positive bacteria, due to its polycationic properties [48]. And, positively charged liposomes inhibit *S*. *aureus* and *P*. *aeruginosa* biofilm formation [49]. Various mechanisms for the inhibition of bacterial biofilm by cationic molecules have been suggested by previous studies. For example, norspermidine alters the expression of gene related with quorum sensing system in *S*. *mutans*, leading to the inhibition of biofilm formation [50]. Water-soluble cationic polymers wrap *S*. *aureus* by electrostatic interaction and turn ζ-potential of *S*. *aureus*, which inhibits the bacterial binding to other bacteria or to surface, resulting in inhibited biofilm formation [51]. Some cationic peptides can penetrate cells to bind to DNA and regulate biofilm-related gene expression in *P*. *aeruginosa* [41]. Therefore, our results, together with those of others, suggest that the D-alanine moiety of LTA is crucial for inhibition of bacterial biofilm formation due to its positive charge.

In the present study, only Lp.LTA inhibited *S*. *mutans* biofilm formation among LTAs from various *Lactobacillus* stains. Accumulating reports suggest that the structural difference of LTAs from various Gram-positive bacteria is responsible for their differential functions [15–18]. LTA from *L*. *plantarum* L-137 strain is known to have 96 repeating units consisting of polyglycerol phosphate backbone (Gro-P) with 50% D-alanine substituent and contain not only di-acylated glycolipid (DAG) but also tri-acylated glycolipid (TAG) [52]. In addition, we also reported that *L*. *plantarum* KCTC10887BP has both DAG and TAG with unsaturated fatty acids [19]. LTA form *L*. *delbrueckii* ATCC15808 strain has 29–37 repeating units consisting of Gro-P with 21–27% D-alanine substituent and contains both DAG and TAG [53]. LTA from *L*. *rhamnosus* GG ATCC53103 strain has 30 repeating units consisting of Gro-P with 71.8% D-alanine substituent and contains only DAG [54]. Thus, the selective inhibitory activity of Lp.LTA could be associated with its molecular structure differing in D-alanine content, length of repeating unit, and glycolipids. Nevertheless, further study is required to clarify it.

*S*. *mutans* is the major bacteria that cause dental caries among various bacterial species present in the oral cavity, and targeting *S*. *mutans* biofilm is critical for the treatment of dental caries. Nevertheless, bacterial biofilms are predominantly formed by multispecies bacteria in oral cavity, which protect them from external environments through cell-to-cell communication, such as physical interaction [55], genetic exchange [56], and diffusible signal [57, 58]. Thus, it is necessary to understand the effect of Lp.LTA on biofilm formation by multispecies bacteria. In order to apply Lp.LTA clinically for the treatment of oral infectious diseases, the effect of Lp.LTA against multispecies bacterial biofilm should be studied.

Lp.LTA inhibited biofilms of *S*. *mutans* laboratory strains and clinical isolates as well. These results imply that Lp.LTA could be widely used to remove or inhibit biofilm formation of various *S*. *mutans* strains. In conclusion, the results of this study suggest that Lp.LTA is crucial for *L*. *plantarum* inhibition of *S*. *mutans* biofilm formation. Lp.LTA could be useful for developing effective therapeutic agents to treat dental infectious diseases caused by *S*. *mutans* biofilms.
